# 1,2-Bis(4-methyl­phen­oxy)ethane

**DOI:** 10.1107/S1600536810042613

**Published:** 2010-10-31

**Authors:** Lu-lu Wang, Wen-ge Yang, Jing Zhu, Kai Wang, Yong-hong Hu

**Affiliations:** a State Key Laboratory of Materials-Oriented Chemical Engineering, School of Pharmaceutical Sciences, Nanjing University of Technology, Xinmofan Road No. 5 Nanjing, Nanjing 210009, People’s Republic of China; b State Key Laboratory of Materials-Oriented Chemical Engineering, College of Life Science and Pharmaceutical Engineering, Nanjing University of Technology, Xinmofan Road No. 5 Nanjing, Nanjing 210009, People’s Republic of China

## Abstract

In the title compound, C_16_H_18_O_2_, the two aromatic rings are almost orthogonal, making a dihedral angle of 89.41 (2)°. There is a C—H⋯π contact between the methyl­ene group and the 4-methyl­phenyl ring. The molecule exhibits twofold symmetry..

## Related literature

For background to the uses of the title compound and further synthetic details, see: Xiao *et al.* (2007 ▶).
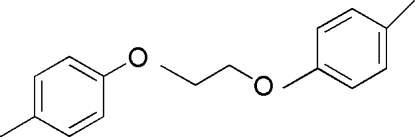


## Experimental

### 

#### Crystal data

C_16_H_18_O_2_
*M* *_r_* = 242.30
Monoclinic, 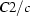

*a* = 27.173 (5) Å
*b* = 5.5510 (11) Å
*c* = 9.2780 (19) Å
β = 93.55 (3)°
*V* = 1396.8 (5) Å^3^
*Z* = 4
Mo *K*α radiation
μ = 0.08 mm^−1^
*T* = 293 K
0.30 × 0.30 × 0.05 mm

#### Data collection

Enraf–Nonius CAD-4 diffractometer
Absorption correction: ψ scan (North *et al.*, 1968 ▶) *T*_min_ = 0.978, *T*_max_ = 0.996
2542 measured reflections
1276 independent reflections
636 reflections with *I* > 2σ(*I*)
*R*_int_ = 0.083
3 standard reflections every 200 reflections  intensity decay: 1%

#### Refinement

*R*[*F* ^2^ > 2σ(*F* ^2^)] = 0.057
*wR*(*F* ^2^) = 0.131
*S* = 1.00
1276 reflections
82 parameters
H-atom parameters constrained
Δρ_max_ = 0.13 e Å^−3^
Δρ_min_ = −0.14 e Å^−3^

### 

Data collection: *CAD-4 EXPRESS* (Enraf–Nonius, 1994 ▶); cell refinement: *CAD-4 EXPRESS*; data reduction: *XCAD4* (Harms & Wocadlo, 1995 ▶); program(s) used to solve structure: *SHELXS97* (Sheldrick, 2008 ▶); program(s) used to refine structure: *SHELXL97* (Sheldrick, 2008 ▶); molecular graphics: *SHELXTL* (Sheldrick, 2008 ▶); software used to prepare material for publication: *PLATON* (Spek, 2009 ▶).

## Supplementary Material

Crystal structure: contains datablocks global, I. DOI: 10.1107/S1600536810042613/ng5032sup1.cif

Structure factors: contains datablocks I. DOI: 10.1107/S1600536810042613/ng5032Isup2.hkl

Additional supplementary materials:  crystallographic information; 3D view; checkCIF report

## Figures and Tables

**Table 1:** Hydrogen-bond geometry (Å, °) *Cg*1 is the centroid of the 4-methylphenyl ring (C1–C6).

*D*—H⋯*A*	*D*—H	H⋯*A*	*D*⋯*A*	*D*—H⋯*A*
C8—H8*A*⋯*Cg*1	0.97	2.85	3.664 (3)	142

**Fig. 1.**
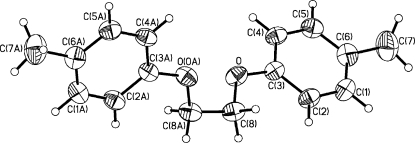
The molecular structure of the title molecule, with the atom numbering scheme. Displacement ellipsoids are drawn at 30% probability levels.

**Fig. 2.**
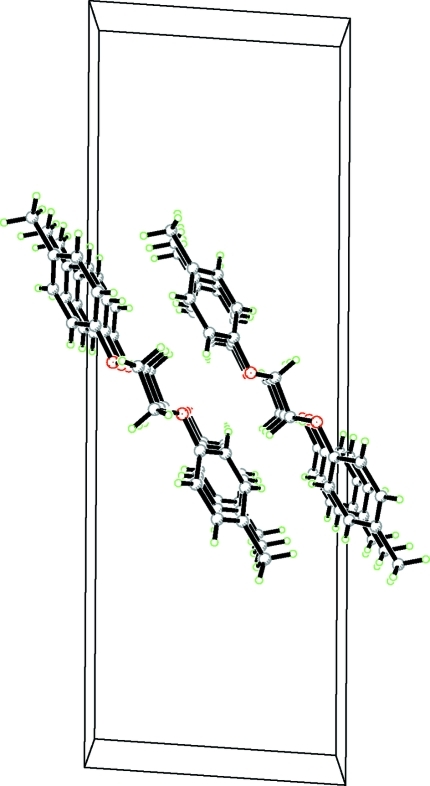
A practical packing diagram of the title compound. There is no intramolecular or intermolecular hydrogen bonds in the crystal.

## supplementary crystallographic information

### Experimental

*p*-Cresol (30.3 g,0.28 mol) was added to a stirred solution of sodium hydroxide(16 g,0.4 mol) in 200 ml of ethanol at room temperature. After stirring for 1 h, ethylene dibromide(28.1 g,0.15 mol) was added. The reaction mixture was stirred and heated under refluxing for another 15 h and then poured into a 5% aqueous solution of NaOH (500 ml). The resulting mixture was cooled to room temperature and filtered. The remaining solid was washed with water(2 *x* 50 ml) and ethanol(2 *x* 40 ml),and then dried *in vacuo* to give the products 13.6 g as white solids (40.1%) (Xiao *et al.*, 2007) Crystals of (I) suitable for X-ray diffraction were obtained by slow evaporation of an ethanol solution.

### Refinement

H atoms were positioned geometrically with C—H = 0.93, 0.98 and 0.97 Å for aromatic, methine and methylene H atoms, respectively, and constrained to ride on their parent atoms, with *U*_iso_(H) = 1.2 (or 1.5 for methyl groups) times *U*_eq_(C).

### Crystal data

**Table:**

C_16_H_18_O_2_	*F*(000) = 520
*M**_r_* = 242.30	*D*_x_ = 1.152 Mg m^−^^3^
Monoclinic, *C*2/*c*	Mo *K*α radiation, λ = 0.71073 Å
*a* = 27.173 (5) Å	Cell parameters from 25 reflections
*b* = 5.5510 (11) Å	θ = 9–12°
*c* = 9.2780 (19) Å	µ = 0.08 mm^−^^1^
β = 93.55 (3)°	*T* = 293 K
*V* = 1396.8 (5) Å^3^	Prism, colorless
*Z* = 4	0.30 × 0.30 × 0.05 mm

### Data collection

**Table:**

Enraf–Nonius CAD-4 diffractometer	636 reflections with *I* > 2σ(*I*)
Radiation source: fine-focus sealed tube	*R*_int_ = 0.083
graphite	θ_max_ = 25.3°, θ_min_ = 1.5°
ω/2θ scans	*h* = −32→32
Absorption correction: ψ scan (North *et al.*, 1968)	*k* = 0→6
*T*_min_ = 0.978, *T*_max_ = 0.996	*l* = −11→11
2542 measured reflections	3 standard reflections every 200 reflections
1276 independent reflections	intensity decay: 1%

### Refinement

**Table:**

Refinement on *F*^2^	Primary atom site location: structure-invariant direct methods
Least-squares matrix: full	Secondary atom site location: difference Fourier map
*R*[*F*^2^ > 2σ(*F*^2^)] = 0.057	Hydrogen site location: inferred from neighbouring sites
*wR*(*F*^2^) = 0.131	H-atom parameters constrained
*S* = 1.00	*w* = 1/[σ^2^(*F*_o_^2^) + (0.022*P*)^2^] where *P* = (*F*_o_^2^ + 2*F*_c_^2^)/3
1276 reflections	(Δ/σ)_max_ < 0.001
82 parameters	Δρ_max_ = 0.13 e Å^−^^3^
0 restraints	Δρ_min_ = −0.14 e Å^−^^3^

### Special details

**Table:**

Geometry. All e.s.d.'s (except the e.s.d. in the dihedral angle between two l.s. planes) are estimated using the full covariance matrix. The cell e.s.d.'s are taken into account individually in the estimation of e.s.d.'s in distances, angles and torsion angles; correlations between e.s.d.'s in cell parameters are only used when they are defined by crystal symmetry. An approximate (isotropic) treatment of cell e.s.d.'s is used for estimating e.s.d.'s involving l.s. planes.
Refinement. Refinement of *F*^2^ against ALL reflections. The weighted *R*-factor *wR* and goodness of fit *S* are based on *F*^2^, conventional *R*-factors *R* are based on *F*, with *F* set to zero for negative *F*^2^. The threshold expression of *F*^2^ > σ(*F*^2^) is used only for calculating *R*-factors(gt) *etc*. and is not relevant to the choice of reflections for refinement. *R*-factors based on *F*^2^ are statistically about twice as large as those based on *F*, and *R*- factors based on ALL data will be even larger.

### Fractional atomic coordinates and isotropic or equivalent isotropic displacement parameters (Å^2^)

**Table:**

	*x*	*y*	*z*	*U*_iso_*/*U*_eq_	
O	0.52863 (6)	0.2044 (3)	0.12559 (15)	0.0667 (6)	
C1	0.65274 (9)	0.1203 (6)	−0.0026 (3)	0.0768 (9)	
H1A	0.6793	0.0147	0.0075	0.092*	
C2	0.61155 (9)	0.0757 (5)	0.0739 (2)	0.0660 (7)	
H2A	0.6105	−0.0578	0.1342	0.079*	
C3	0.57211 (9)	0.2313 (5)	0.0597 (2)	0.0543 (6)	
C4	0.57452 (9)	0.4268 (5)	−0.0305 (2)	0.0635 (7)	
H4A	0.5479	0.5321	−0.0411	0.076*	
C5	0.61569 (10)	0.4681 (5)	−0.1049 (3)	0.0704 (8)	
H5A	0.6166	0.6022	−0.1647	0.085*	
C6	0.65576 (10)	0.3158 (6)	−0.0932 (3)	0.0752 (9)	
C7	0.70126 (10)	0.3646 (6)	−0.1759 (3)	0.1183 (13)	
H7A	0.7254	0.2418	−0.1539	0.177*	
H7B	0.7147	0.5190	−0.1485	0.177*	
H7C	0.6924	0.3637	−0.2777	0.177*	
C8	0.52487 (8)	0.0048 (5)	0.2210 (2)	0.0663 (8)	
H8A	0.5499	0.0169	0.2998	0.080*	
H8B	0.5300	−0.1444	0.1696	0.080*	

### Atomic displacement parameters (Å^2^)

**Table:**

	*U*^11^	*U*^22^	*U*^33^	*U*^12^	*U*^13^	*U*^23^
O	0.0811 (12)	0.0665 (12)	0.0532 (9)	0.0140 (11)	0.0085 (9)	0.0125 (11)
C1	0.0708 (18)	0.082 (2)	0.0774 (18)	0.0161 (17)	0.0044 (15)	−0.001 (2)
C2	0.0797 (17)	0.0638 (18)	0.0538 (14)	0.0115 (17)	−0.0030 (13)	0.0044 (16)
C3	0.0670 (16)	0.0574 (16)	0.0382 (12)	0.0066 (15)	−0.0003 (12)	−0.0046 (13)
C4	0.0806 (18)	0.0546 (17)	0.0548 (13)	0.0090 (15)	0.0012 (13)	0.0019 (16)
C5	0.0858 (19)	0.0658 (19)	0.0595 (15)	−0.0035 (17)	0.0023 (15)	0.0060 (17)
C6	0.0771 (19)	0.087 (2)	0.0617 (16)	−0.0028 (19)	0.0093 (15)	−0.006 (2)
C7	0.088 (2)	0.152 (4)	0.118 (2)	−0.005 (2)	0.0279 (19)	0.008 (3)
C8	0.0914 (19)	0.0611 (16)	0.0462 (12)	0.0077 (15)	0.0023 (12)	0.0064 (14)

### Geometric parameters (Å, °)

**Table:**

O—C3	1.372 (2)	C5—C6	1.377 (4)
O—C8	1.426 (3)	C5—H5A	0.9300
C1—C6	1.378 (4)	C6—C7	1.519 (3)
C1—C2	1.384 (3)	C7—H7A	0.9600
C1—H1A	0.9300	C7—H7B	0.9600
C2—C3	1.376 (3)	C7—H7C	0.9600
C2—H2A	0.9300	C8—C8^i^	1.485 (4)
C3—C4	1.375 (3)	C8—H8A	0.9700
C4—C5	1.370 (3)	C8—H8B	0.9700
C4—H4A	0.9300		
			
C3—O—C8	117.25 (18)	C1—C6—C5	117.0 (3)
C6—C1—C2	122.3 (3)	C1—C6—C7	122.0 (3)
C6—C1—H1A	118.9	C5—C6—C7	121.0 (3)
C2—C1—H1A	118.9	C6—C7—H7A	109.5
C3—C2—C1	119.2 (3)	C6—C7—H7B	109.5
C3—C2—H2A	120.4	H7A—C7—H7B	109.5
C1—C2—H2A	120.4	C6—C7—H7C	109.5
O—C3—C4	115.6 (2)	H7A—C7—H7C	109.5
O—C3—C2	125.2 (2)	H7B—C7—H7C	109.5
C4—C3—C2	119.2 (2)	O—C8—C8^i^	109.05 (18)
C5—C4—C3	120.7 (3)	O—C8—H8A	109.9
C5—C4—H4A	119.7	C8^i^—C8—H8A	109.9
C3—C4—H4A	119.7	O—C8—H8B	109.9
C4—C5—C6	121.6 (3)	C8^i^—C8—H8B	109.9
C4—C5—H5A	119.2	H8A—C8—H8B	108.3
C6—C5—H5A	119.2		
			
C6—C1—C2—C3	−0.1 (4)	C3—C4—C5—C6	0.5 (4)
C8—O—C3—C4	−179.46 (19)	C2—C1—C6—C5	0.1 (4)
C8—O—C3—C2	2.8 (3)	C2—C1—C6—C7	179.5 (2)
C1—C2—C3—O	177.9 (2)	C4—C5—C6—C1	−0.3 (4)
C1—C2—C3—C4	0.2 (3)	C4—C5—C6—C7	−179.7 (3)
O—C3—C4—C5	−178.3 (2)	C3—O—C8—C8^i^	−179.03 (19)
C2—C3—C4—C5	−0.4 (3)		

Symmetry codes: (i) −*x*+1, *y*, −*z*+1/2.

### Hydrogen-bond geometry (Å, °)

**Table:**

*D*—H···*A*	*D*—H	H···*A*	*D*···*A*	*D*—H···*A*
C8—H8A···Cg1	0.97	2.85	3.664 (3)	142
